# Social Determinants of Cognitive and Brain Aging: Novel Approaches and New Findings

**DOI:** 10.1093/geroni/igaf122.741

**Published:** 2025-12-31

**Authors:** Andrea Rosso, Lisa Barnes

## Abstract

Social determinants of health, including socioeconomic status (SES), neighborhood conditions and discrimination, are now recognized as critical modifiable risk factors for poor cognitive and brain aging. However, there remain a number of uncertainties regarding their role, including specific pathways and important aspects of exposure. Here, we explore some of these questions by interrogating exposure definitions, employing a lifecourse perspective, exploring study design effects on findings, and testing specific mediation pathways. We incorporate data from 5 cohorts, spanning local neighborhood studies to nationally-representative samples, to explore these questions. First, Meredith Phillips assesses how subjective and objective measures of SES relate to neuroimaging markers of brain health in a middle-aged cohort to address which aspects of SES are most critical to brain health. Second, Andrea Rosso examines neighborhood deprivation measures across the lifecourse in relation to general cognitive aging in a cohort based in 2 historically Black neighborhoods to address critical periods of exposure. Third, Erica Fan assesses the relation of everyday discrimination in relation to cognitive tests across 3 cohorts, exploring how study design and race may affect results. Finally, Greta Cheng tests a mediation pathway from neighborhood SES to general cognitive function through hemoglobin A1C in the Health and Retirement Study. Together, these results advance our understanding of the role of social determinants in cognitive and brain aging with implications for study design and methods. A discussion led by Lisa Barnes will address the advances presented in these studies and consider next steps.

